# Genomic structure and expression of the human serotonin 2A receptor gene (*HTR2A*) locus: identification of novel *HTR2A* and antisense (*HTR2A-AS1*) exons

**DOI:** 10.1186/s12863-015-0325-6

**Published:** 2016-01-06

**Authors:** Cara L. Ruble, Ryan M. Smith, John Calley, Leanne Munsie, David C. Airey, Yuan Gao, Joo Heon Shin, Thomas M. Hyde, Richard E. Straub, Daniel R. Weinberger, Laura K. Nisenbaum

**Affiliations:** Tailored Therapeutics, Lilly Corporate Center, Eli Lilly and Company, Indianapolis, IN ᅟ; Lieber Institute for Brain Development, Baltimore, MD ᅟ; Departments of Neurology, Psychiatry and Behavioral Sciences, John Hopkins University School of Medicine, Baltimore, MD ᅟ; Departments of Psychiatry, Neurology, Neuroscience, and the Institute of Genetic Medicine, Johns Hopkins University School of Medicine, Baltimore, MD ᅟ

**Keywords:** Serotonin, HTR2A, Alternative splicing, Antisense RNA, mRNA expression, Regulatory polymorphism, Allelic expression, Comparative genomics, Long non-coding RNA, 5-HT2A

## Abstract

**Background:**

The serotonin 2A receptor is widely implicated in genetic association studies and remains an important drug target for psychiatric, neurological, and cardiovascular conditions. RNA sequencing redefined the architecture of the serotonin 2A receptor gene (*HTR2A*), revealing novel mRNA transcript isoforms utilizing unannotated untranslated regions of the gene. Expression of these untranslated regions is modulated by common single nucleotide polymorphisms (SNPs), namely rs6311. Previous studies did not fully capture the complexity of the sense- and antisense-encoded transcripts with respect to novel exons in the *HTR2A* gene locus. Here, we comprehensively catalogued exons and RNA isoforms for both *HTR2A* and *HTR2A-AS1* using RNA-Seq from human prefrontal cortex and multiple mouse tissues. We subsequently tested associations between expression of newfound gene features and common SNPs in humans.

**Results:**

We find that the human *HTR2A* gene spans ~66 kilobases and consists of 7, rather than 4 exons. Furthermore, the revised human *HTR2A-AS1* gene spans ~474 kilobases and consists of 18, rather than 3 exons. Three *HTR2A* exons directly overlap with *HTR2A-AS1* exons, suggesting potential for complementary nucleotide interactions. The repertoire of possible mouse *Htr2a* splice isoforms is remarkably similar to humans and we also find evidence for overlapping sense-antisense transcripts in the same relative positions as the human transcripts. rs6311 and SNPs in high linkage disequilibrium are associated with *HTR2A-AS1* expression, in addition to previously described associations with expression of the extended 5’ untranslated region of *HTR2A*.

**Conclusions:**

Our proposed *HTR2A* and *HTR2A-AS1* gene structures dramatically differ from current annotations, now including overlapping exons on the sense and anti-sense strands. We also find orthologous transcript isoforms expressed in mice, providing opportunities to elucidate the biological roles of the human isoforms using a model system. Associations between rs6311 and expression of *HTR2A* and *HTR2A-AS1* suggest this polymorphism is capable of modulating the expression of the sense or antisense transcripts. Still unclear is whether these SNPs act directly on the expression of the sense or antisense transcripts and whether overlapping exons are capable of interacting through complimentary base-pairing. Additional studies are necessary to determine the extent and nature of interactions between the SNPs and the transcripts prior to interpreting these findings in the context of phenotypes associated with *HTR2A*.

**Electronic supplementary material:**

The online version of this article (doi:10.1186/s12863-015-0325-6) contains supplementary material, which is available to authorized users.

## Background

The serotonin 2A receptor (5-HT_2A_) is a G protein-coupled receptor (GPCR) that serves as a primary target for serotonin signaling and is expressed on many cell types in the brain and periphery. 5-HT_2A_ remains of great scientific interest due to its many roles in normal biological functions, which include cerebral cortex excitability [1], platelet aggregation [2], smooth muscle contraction [3], vasoconstriction and dilation [4], inflammatory processes [5], and hormone signaling [6]. Among the many tissues expressing 5-HT_2A_, it is especially prevalent in the cerebral cortex, enriched at the apical dendrites of pyramidal neurons [7, 8]. Commensurate with its broad biological influence, studies examining single nucleotide polymorphisms (SNPs) in the gene encoding 5-HT_2A_ (*HTR2A*) have identified more than 100 genotypic associations with a wide range of phenotypes, especially brain-related disorders [9]. Drugs that directly or indirectly modulate serotonergic signaling through 5-HT_2A_ receptors are used to treat neuropsychiatric, neurologic, and cardiovascular conditions, and 5-HT_2A_ is an emerging drug target for a variety of other indications [10–13].

Transcriptome profiling in human tissues has greatly increased our appreciation for RNA isoform diversity, revealing pervasive alternative splicing and transcription start and termination sites for nearly all multi-exon genes [14, 15]. Consequently, we can utilize this information to better understand disease states and tailor therapeutics [16], but this requires in-depth characterization of RNA expression for important targets, such as *HTR2A*. Knowledge of the different exons and mRNAs for *HTR2A* has evolved over the past 20 years. Human *HTR2A* complementary DNA (cDNA) was first cloned in 1991 (GenBank X57830.1) [17], followed in 1992 by a genomic structure consisting of 3 exons spanning 20kB [18]. The original Reference Sequence (RefSeq) mRNA annotation (NM_000621.1) reflects these findings. Since this first annotation, *HTR2A* has undergone three revisions, which removed cloning sites and altered alleles at polymorphic sites (NM_000621.2), added a 5’ exon (NM_000621.3), and extended the 3’ untranslated region (UTR), adding a consensus polyadenylation signal (NM_000621.4). The current gene model consists of 4 exons, spanning more than 65kB, and also includes evidence for alternative splicing of exon 2 (NM_001165947). Based on length, these two RefSeq annotations (NM_000621.4 and NM_001165947.2) likely reflect the transcripts detected by Northern blot in 1990 [19]. There are additional *HTR2A* splice variants described in the literature that are not yet annotated in RefSeq. This includes an RNA isoform containing a novel 118-bp exon residing in intron 3 of the current annotation [20] and four additional exon boundary changes resulting in novel RNA isoforms: a truncated exon 2, a retained intron between exons 1 and 2, a 5’ extension of exon 1, and a 3’ extension of exon 4 [21].

The *HTR2A* locus also hosts a long non-coding RNA (lncRNA) gene on the antisense strand, annotated as *HTR2A-AS1* in RefSeq, and encoding two alternatively spliced isoforms (NR_103752 and NR_046612). Four Expressed Sequence Tags (ESTs) (AI076014.1, AI216351.1, AI914390.1, and AW469493.1) suggest the existence of at least three different splice variants, some of which are detectable following PCR amplification of cDNA synthesized from human brain and testes [21, 22]. Furthermore, *HTR2A-AS1* expression is readily detectable in human testes according to RNA-Seq data from the Genotype-Tissue Expression (GTEx) project [23]. However, expression is sparse in other GTEx-investigated tissues, including the cerebral cortex (www.GTExportal.org).

The 5-HT_2A_ gene in mice spans approximately 66kB on chromosome 14 and contains 3 exons. The mouse gene shares many similarities with the human gene. For example, the relative genomic spacing of the protein-coding exons and the use of constitutive splice sites is conserved, such that the mouse exon 1/human exon 2 both encode the first 138 amino acids of 5-HT2A, followed by a relatively short intron (2.6kB in mouse, 2.9kB in human). Mouse exon 2/human exon 3 both encode the subsequent 67 amino acids, followed by a relatively large intron (60.4kB in mouse, 56.8kB in humans). Mouse exon 3/human exon 4 both encode the remaining 266 amino acids. However, mouse *Htr2a* currently lacks in-depth analysis of RNA isoform expression to delineate alternative transcripts, as previously performed on human *HTR2A*. Therefore, it is unclear how well the mouse recapitulates the molecular biology of human *HTR2A* splice variants, limiting its capacity as a model organism for understanding *HTR2A* expression.

At the crux of the many genotype-phenotype associations involving *HTR2A* is whether the implicated genetic variants have functional consequences. The most frequently cited SNPs in studies concerning *HTR2A* are rs6311 and rs6313. rs6311 and rs6313 are in near-perfect linkage disequilibrium (LD) [24], located 1538 bases apart on chromosome 13, and neither alter the encoded protein. rs6311 (also known as -1438G > A and A-1438G) is historically described as being located in the upstream or promoter region of *HTR2A*. However, rs6311 is transcribed in minor isoforms of *HTR2A* mRNA expressed in the brain, perhaps through the use of an alternative transcription start site that results in an extended 5’ UTR [21]. The variant “A” allele of rs6311 is associated with reduced expression of isoforms containing this 5’ UTR extension. rs6311 has also been correlated with total *HTR2A* mRNA and protein expression [25, 26], although multiple studies failed to find an allele-specific effect of rs6311 on the most abundant primary transcript isoform [21, 27, 28]. rs6313 (also known as T102C) is a synonymous SNP residing in exon two. Because rs6313 lacks strong functional evidence and is in high LD with rs6311, genetic associations with rs6313 are often attributed to the functional consequences of rs6311.

Understanding the functional consequences of SNPs is a critical first step towards appreciating their roles in disease. To date, only a single study has examined *HTR2A* SNPs and the expression of the extended 5’ UTR isoform in affected tissues, finding no difference between post-mortem brain tissue from autistic individuals and controls [29]. To better understand the role of *HTR2A* in human disease, here we have comprehensively catalogued the transcripts expressed from the *HTR2A* gene locus in the dorsolateral prefrontal cortex (DLPFC) from unaffected controls and schizophrenia patients. We also characterized *Htr2a* locus expression in the mouse, contrasting it with our human findings to determine where mice could provide models for studying human 5-HT_2A_ function. Following, we asked whether rs6311 and rs6313 are associated with sense or antisense transcript expression.

## Results

### *HTR2A* exons defined by splice junction analysis

**Fig. 1:**
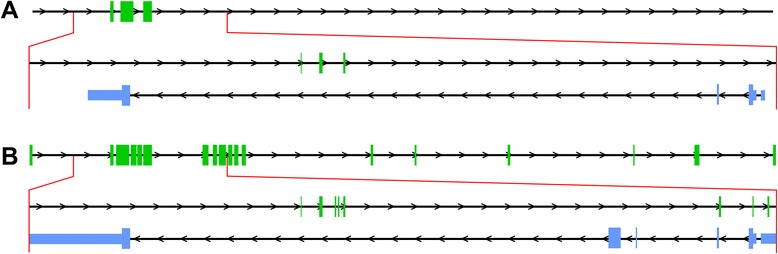
Human *HTR2A-AS1* (green) and *HTR2A* (blue) gene locus (chr13:47,370,678-47,844,384). **a** The current RefSeq annotation for *HTR2A-AS1* includes three exons located within the third intron of *HTR2A*. **b** Splice junction analysis expands the number of exons for both *HTR2A-AS1* to include 18 total exons, three of which overlap with exons at the 5’ end of *HTR2A* (see Fig. 3). We also found evidence that supports extensions of both untranslated regions, represented by the thinner blue bars at the terminal ends of the gene, and the addition of two exons (3a and 3b) for *HTR2A. Note*: The panoramic view of *HTR2A-AS1*, represented on the first lines of figures A and B, have exons enlarged approximately 30-fold relative to introns. The magnified loci between the red lines have exons and introns represented at a 1:1 scale. Arrows indicate the direction of transcription

In addition to the four annotated exons, we found evidence for four different alternative exons, including two that have not been previously described (exons 0 and 3b; Table 1). The revised gene model is presented in Fig. 1b. The majority of exon-exon junction reads join exons from previously annotated isoforms. However, novel exons also share splice junctions with the previously annotated exons, suggesting that they are included in mature RNA transcripts (Table 2; Additional file 1: Figure S1). The strength of predicted splice donor/acceptor sites and branch point sequences, assessed *in silico* using the Human Splicing Finder (http://www.umd.be/HSF/) [30], do not significantly differ across the novel and annotated exons (Additional file 2: Figure S2a). Furthermore, the stretch of nucleotides 5 to 40 bases upstream of the novel exons, presumably the polypyrimidine tracts, are more highly enriched for pyrimidine and uracil nucleotides relative to the annotated exons (Additional file 2:Figure S2a). We also used the Alternative Splice Site Predictor (http://wangcomputing.com/assp/index.html) [31] to identify and score any possible splice donor and acceptor site across the entire *HTR2A* locus. We subsequently compared scores at annotated and novel exons versus all other predicted donor and acceptor sites in *HTR2A*. Again, we found that average scores for the novel exons did not significantly differ from the annotated exons, which both scored higher than all other predicted donor/acceptor sites (Additional file 2: Figure S2b).

**Table 1:** *HTR2A* Exons

Exon	Size (bp)	Coordinates^a^	Notes
0	187	chr13:47472087-47472273	Newly described
1ext	1465	chr13:47470809-47472273	Described in Smith et al., 2013
1int	2644	chr13:47469630-47472273	Described in Smith et al., 2013
1	403	chr13:47470809-47471211	As annotated in RefSeq
2	740	chr13:47469630-47469825	As annotated in RefSeq
2tr	196	chr13:47469630-47470369	Described in Smith et al., 2013
3	201	chr13:47466525-47466725	As annotated in RefSeq
3a	118	chr13:47458674-47458791	Described in Guest et al., 2000
3b	1175	chr13:47456022-47457197	Newly described
4	4098	chr13:47405677-47409774	As annotated in RefSeq
4ext	9716	chr13:47400058-47409774	Partially described in Smith et al., 2013

^a^Coordinates reflect hg19/GRCh37

**Table 2:** *HTR2A* Splice Junctions

Splices From:	Splices To:	Total Junction Counts^a^	Samples Observed
0	2	28	21
0	2tr	1	1
0	3	126	73
0	4	1	1
1 or 1ext	2	15802	205
1 or 1ext	2tr	45	39
1 or 1ext	3	16	14
1 or 1ext	3b	1	1
2 or 1int	3	15253	200
2 or 1int	3b	1	1
3	3a	72	54
3	3b	39	34
3	4 or 4ext	14221	203
3a	3b	26	19
3a	4 or 4ext	108	60

^a^Summed across all samples

Exons 1, 1int, 1ext, and 0 do not have exon-exon junctions defining their 5’ boundaries and are therefore considered as transcription initiation sites. The parsimonious interpretation based on RNA-Seq coverage in this area supports the existence of two transcription start sites. Transcripts containing exons 0, 1ext, and at least some of 1int likely use a transcription initiation site upstream of the annotated exon 1, consistent with previous reports [21]. However, the majority of transcripts likely utilize the transcription initiation site beginning with exon 1, given the large increase in read depth at this exon (Additional file 3: Figure S3). Exons 3b and 4 do not have exon-exon junctions defining their 3’ boundaries. Exon 4 contains a canonical poly-adenylation (poly-A) signal (AATAAA) at position chr13:47405698-47405703, after which the number of mapped reads drops off precipitously, signaling the termination of the major transcript isoform(s) (Additional file 4: Figure S4). Consistent with previous findings, we observed a continuation of the 3’ UTR that extends beyond the canonical poly-A signal [21], continuing 5.6kB distally from the annotated 3’ UTR to a cluster of four canonical poly-A signals within a stretch of 88 nucleotides (chr13:47400058-47400144; Additional file 4: Figure S4). The lack of an exon-exon junction read to define the 3’ boundary of exon 3b suggests that it is unlikely to be spliced into transcripts containing exon 4 and could represent a terminal exon. Reduced read coverage following a cluster of canonical and non-canonical poly-A signals supports the interpretation that exon 3b is a terminal exon (Additional file 5: Figure S5).

### Transcript assembly for *HTR2A*

We assembled likely mRNA isoforms, excluding junctions where we see only single junction counts. We then assessed the predicted open reading frames and calculated the number of predicted transmembrane (TM) domains (Table 3). Only transcript isoforms utilizing exons 2, 3, and 4 appear capable of translating protein isoforms with all 7TM domains quintessential of GPCRs.

**Table 3:** Putative Transcript Assembly for *HTR2A*

mRNA Exons Utilized	# of Predicted TM Domains
1^2^3^4	7
0^2^3^4	7
1^2tr^3^4	6
0^3^4	5
1^3^4	5
1^2^3^3b	3
1^2^3^3a^4	3
0^3^3a^4	3
0^3^3b	No ORF

### *HTR2A-AS1* exons defined by splice junction analysis

*HTR2A-AS1* is on the forward strand of chromosome 13. Definition of *HTR2A-AS1* splice junctions followed the same rules regarding canonical splicing as applied to *HTR2A*, allowing for stranded assignment of junction reads. We first examined the transcriptome for evidence of junction reads for the annotated *HTR2A-AS1* gene. Only the annotated exon 2 was identified through an exon junction read in this dataset. However, our analysis of exon-exon junction reads across canonical exon boundaries defined on the forward strand, agnostic to annotated *HTR2A-AS1* exons, identified 15 additional exons. Four of these exons undergo apparent alternative splicing, resulting in a total of 22 discretely defined exons (Fig. 1b; Table 4). The frequency of these exon junction reads varied widely (Additional file 6: Table S2), suggesting varying abundances of many different *HTR2A-AS1* splice isoforms. Notably, multiple antisense exons overlapped sense-encoded *HTR2A* exons (Fig. 3a). Exons 1, 2, 4, 5, 8, 9, 9.1, 10, 11, and 12 did not have an exon junction read defining their 5’ boundary, so we presume them to be transcription start sites. For these exons, we have used Eponine [32] to predict and score putative transcription start sites (Table 4). However, it is also possible that these exons represent internal exons, and we do not have adequate depth to capture all exon-exon junctions, especially for exons with low expression. One canonical poly-A signal was observed in exon 18.

**Table 4:** *HTR2A-AS1* Exons

Exon	Size	Coordinates (hg19)^a^	Predicted TSS^b^ (hg19)	TSS Score^b^	Notes
1	71^c^	chr13:47370678-47370748	chr13:47370715-47370719	0.8972	
2	82^c^	chr13:47426280-47426361	chr13:47426295-47426299	0.5747	As annotated in RefSeq
3	316	chr13:47428074-47428389			As annotated in RefSeq; No junction reads observed
3.1	144	chr13:47428074-47428217			
4	139^c^	chr13:47429408-47429546	chr13:47429250-47429253	0.2663	
5	117^c^	chr13:47429726-47429842	chr13:47429797-47429804	0.5568	
6	210	chr13:47430225-47430434			As annotated in RefSeq; No junction reads observed
7	133	chr13:47466546-47466678			Overlaps *HTR2A* exon 3
7.1	194	chr13:47466546-47466739			Overlaps *HTR2A* exon 3
8	101^c^	chr13:47469779-47469879	chr13:47469614-47469622	0.7864	Overlaps *HTR2A* exon 2
9	167^c^	chr13:47471212-47471378	chr13:47471046-47471066	0.9737	Overlaps *HTR2A* exon 1ext
9.1	101	chr13:47471282-47471382			Overlaps *HTR2A* exon 1ext
10	101^c^	chr13:47472971-47473071	chr13:47473021-47473029	0.7151	
11	101^c^	chr13:47474379-47474479	chr13:47474236-47474243	0.7058	
12	101^c^	chr13:47476850-47476950	chr13:47476912-47476920	0.7333	
13	58	chr13:47566052-47566109			
14	46	chr13:47595782-47595827			
15	59	chr13:47660942-47661000			
16	29	chr13:47748905-47748933			
17	123	chr13:47791699-47791821			
17.1	119	chr13:47791703-47791821			
18	69	chr13:47844316-47844384			Contains canonical polyA signal

^a^Coordinates reflect hg19/GRCh37

^b^Scores are predicted by Eponine (Down & Hubbard, 2002) and includes 200 bp of upstream sequence. Only the highest score for each exon is reported

^c^No 5’ exon junction is defined, so size is estimated based on local read depth

### Transcript assembly for *HTR2A-AS1*

From the 22 different *HTR2A-AS1* exons we found to be expressed in the DLPFC, we inferred the transcript isoforms by examining the number of incoming junction reads spliced from upstream exons versus the outgoing junction reads going to downstream exons. Furthermore, we grouped junctions according to how often they were observed (number of samples, number of junction reads) into rare, low, medium, and high frequency groups, to aid our inference of transcript isoforms (Additional file 6: Table S2). Rare classification required only a single junction read in a single sample. Low, medium, and high classifications required a minimum of 2 reads in 2 samples, 25 reads in 25 samples, and 100 reads in 100 samples, respectively. Next, we exhaustively assembled 57 models of possible *HTR2A-AS1* RNA isoforms, which begin at transcription start sites, allow for only a single low coverage exon junction, and terminate at exons 17 or 18 (Additional file 7: Table S3 and HTR2A-AS1.FASTA). While we find it unlikely that 57 different transcripts are expressed from the *HTR2A-AS1* locus, we used permissive criteria for assembly to next test for protein-coding potential of any possible antisense transcript. The transcript isoform models range in size from 248 to 626 nt. From all possible transcripts, the longest predicted open reading frame (ATG-to-stop) is only 31 amino acids long and is not supported as coding using the tcode program in EMBOSS [33]. Therefore, we find it likely that *HTR2A-AS1* represents an lncRNA.

### Mouse *Htr2a* and *Htr2a-AS1* alignments

Using publically available RNA-Seq data generated from a variety of mouse tissues (Gene Expression Omnibus; GEO Accessions GSE36025, GSE52564, and GSE27243) [34–36], we analyzed mouse *Htr2a* and *Htr2a-AS1* expression in a manner similar to the human brain tissues. The majority of reads mapped to the three annotated exons for *Htr2a*. However, we also found evidence for seven additional alternative sense-encoded exons (Additional file 8: Table S4). In the mouse, *Htr2a* exons 1 and 2 can be alternatively spliced to form low-abundance isoforms orthologous to human transcripts (Additional file 9: Figure S6). Of particular note, the highly-expressed archetype mouse isoform is orthologous to the human isoform that retains intron 1. The mouse also expresses an orthologue to the human isoform in which exon 2 is spliced out. This is predicted to translate a protein that lacks the first two transmembrane domains, consistent with the human isoform lacking exon 2. We also observed infrequent utilization of the splice acceptor site equivalent to the human exon 2 truncation (E2^tr^) that truncates the *N*-terminus of 5-HT_2A_, resulting in a 6TM isoform. However, the splice donor site in mouse differs from that in humans, resulting in a predicted protein that includes all seven transmembrane domains, but lacks 71 of 75 amino acids constituting the *N*-terminus. Finally, much like human *HTR2A*, the mouse gene has a well-expressed extended 3’ UTR, approximately 2.6kB longer than the current annotation, terminating at a canonical poly-A site (Additional file 10: Figure S7). The lack of a poly-A site (canonical or non-canonical) at the end of the current annotation and the contiguous high expression into the extended 3’ UTR argues for revised annotation. As noted in Additional file 8: Table S4 and Additional file 9: Figure S6, we also see alternative splicing of the 3’ UTR, which is predicted to shorten the protein by a single amino acid and change three terminal amino acids (*…NEKVSCV* vs. *…NEKMPF*). This alternative isoform also excludes a large portion of the 3’ UTR, the significance of which is unknown. Unlike human *HTR2A*, we do not see strong evidence for an extended 5’ UTR in the mouse.

**Fig. 2:**
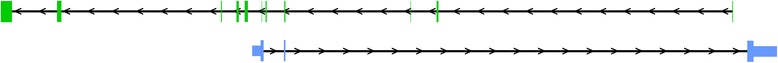
Mouse *Htr2a-AS1* (green) and *Htr2a* (blue) gene locus (chr14: 74,607,947- 74,709,473). Currently, there is no mouse annotation for *Htr2a-AS1* and the RefSeq annotation for *Htr2a* includes a shortened 3’ UTR as compared to gene model presented here (see Additional file 5: Figure S5). Similar to human *HTR2A*, the antisense transcript in mouse overlaps *Htr2a* exons. *Note*: arrows indicate the direction of transcription

**Fig. 3:**
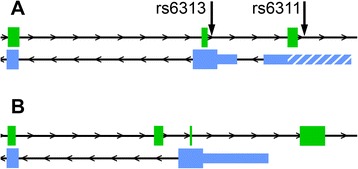
Overlapping *HTR2A* (blue) and *HTR2A-AS1* (green) exons in human (A) and mouse (B). **a** Antisense exons 7, 8, and 9 overlap human *HTR2A* exons 1, 2, and 3. Antisense exons 7 and 9 undergo alternative splicing, resulting in 5 unique exons that can overlap *HTR2A* transcripts. **b** Mouse antisense exons are situated similarly to the human antisense exons. *Note*: orientation of the mouse genes are reversed and mouse *Htr2a* exon 2 is aligned with human *HTR2A* exon 3 for comparison to the human genes. Arrows indicate the direction of transcription. Thin blue bars represent untranslated regions, with the hatched region of the human gene representing the extended 5’ UTR

The mouse alignments suggest the presence of a low-abundance antisense transcript, extending approximately 96 kB and comprised of up to 11 exons (Additional file 11: Table S5; Fig. 2). This antisense transcript also shares similarities to human *HTR2A-AS1*. In particular, the mouse expresses antisense isoforms that overlap sense-encoded exons of *Htr2a* and at similar positions as the human transcripts (Fig. 3b). The functional role for the antisense in regulating *Htr2a* function, if any, is not clear. However, the similarities in overlapping sense and antisense exons between mouse and human tissue lend support for a conserved regulatory function.

### Genetic influence of *HTR2A* and *HTR2A-AS1* expression

Guided by our newfound understanding of *HTR2A* and *HTR2A-AS1* gene structure, we tested the effects of the phenotype-associated SNPs rs6311 and rs6313 on various aspects of expression. To begin, we measured allelic RNA expression to identify influences of *cis*-acting genetic variants. Specifically, we tested whether rs6311 confers an allele-specific influence on the expression of the extended 5’ UTR. Previously, the ancestral “G” allele was associated with increased expression relative to the variant “A” allele [21, 29]. Eighty-two of 109 DLPFC samples heterozygous rs6311had high-quality base calls over the rs6311 position, but low coverage precluded allelic analysis on individual samples. Instead we combined all of the reads at the rs6311 position across the 82 samples, observing significantly more reads of the “G” allele relative to the “A” allele (187 vs 109 counts; binomial test *p* = 0.0004), consistent with previous studies [21, 29]. We further tested whether rs6313 or rs6311 influenced expression of the highly expressed primary splice isoform in an allele-specific manner by measuring allelic expression at rs6313. Greater coverage at rs6313 allowed us to examine allelic expression directly at this SNP in individual samples, rather than relying on pooled counts across samples. If rs6311 or rs6313 were responsible for driving allele-specific expression of the primary transcript, our analysis should reveal imbalanced expression in most, if not all samples. However, only 9 of 109 samples heterozygous for rs6313 demonstrated significantly imbalanced expression ranging from 1.83 to 2.87-fold differences in expression across alleles (Table 5). The deviations from balanced expression suggest the presence of additional low frequency *cis*-acting regulatory variants, but argue strongly against a role for rs6311 or rs6313 in modulating expression of the highly-abundant *HTR2A* mRNA isoform lacking the extended 5’ UTR.

**Table 5:** Significant Allelic Expression Imbalance at rs6313

Sample	Diagnosis	G alleles	A alleles	Binom Dist *p*-val	Corrected *p*-val
1	Scz	92	36	3.94E-07	4.30E-05
2	Scz	79	31	2.67E-06	2.91E-04
3	Control	143	78	7.31E-06	7.96E-04
4	Scz	95	48	5.25E-05	5.72E-03
5	Scz	46	16	8.82E-05	9.61E-03
6	Scz	67	30	1.09E-04	1.19E-02
7	Control	69	32	1.48E-04	1.61E-02
8	Control	57	26	4.39E-04	4.78E-02
9	Scz	78	41	4.44E-04	4.84E-02

In addition to direct allelic expression measures, we tested whether rs6311 or rs6313 are significantly associated with other *HTR2A* and *HTR2A-AS1* expression measures, analogous to expression quantitative trait loci (eQTL). This included uniquely mapped reads covering all *HTR2A* or *HTR2A-AS1* exons, reads aligned to specific exons, and junction reads for both *HTR2A* and *HTR2A-AS1* in the total DLPFC sample cohort. These analyses revealed no significant genotype-expression associations for *HTR2A* prior to Bonferroni correction. However, multiple aspects of *HTR2A-AS1* expression were significantly associated with rs6311 and rs6313 genotypes, including total unique *HTR2A-AS1* reads, *HTR2A-AS1* exon 14 and 17.1 reads, and exon 14–17.1 junction reads (summarized in Table 6). Only the genotypic effect on exon 14 reads remained significant after strict Bonferroni correction. In all instances, increased expression of *HTR2A-AS1* is associated with the variant alleles of rs6311 and rs6313 (A and T, respectively).

**Table 6:** Associations Between rs6311 or rs6313 and RNA Expression

	rs6311					rs6313				
Gene	G/G (*n*)	G/A (*n*)	A/A (*n*)	*P*-Value^a^	Adjusted P-Value	C/C (*n*)	C/T (*n*)	T/T (*n*)	*P*-Value^a^	Adjusted *P*-Value
*HTR2A-AS1* Unique Reads^b^	34.7 (69)	42.0 (106)	40.8 (26)	*0.0070*	0.4204	35.1 (73)	42.0 (103)	40.3 (25)	*0.0153*	0.9163
*HTR2A* Unique Reads^c^	4305.0 (71)	4525.7 (111)	3856.6 (26)	0.3026	1.0000	4262.2 (75)	4571.2 (108)	3934.5 (25)	0.3203	1.0000
*HTR2A, AS1* Overlapping Reads^d^	69.9 (71)	75.8 (111)	62.0 (26)	0.3595	1.0000	69.2 (75)	75.8 (108)	63.9 (25)	0.4112	1.0000
Exon										
*HTR2A-AS1* Exon 14 Reads^e^	13.2 (71)	17.2 (111)	16.7 (26)	*0.0004*	0.0212	13.4 (75)	17.2 (108)	16.6 (25)	*0.0010*	*0.0586*
*HTR2A-AS1* Exon 17.1 Reads^f^	16.4 (69)	21.3 (106)	19.6 (26)	*0.0011*	*0.0687*	16.6 (73)	21.3 (103)	19.6 (25)	*0.0030*	0.1771
Junction Reads										
*HTR2A-AS1* Exon 14 to 17.1 Reads^g^	7.9 (69)	10.3 (108)	9.8 (27)	*0.0069*	0.4169	8.1 (73)	10.2 (105)	9.8 (26)	*0.0142*	0.8532

Count values in the table are model predicted least square means. Differences in the subject numbers across outcomes reflect missing data in the selected variables for each model

^a^Calculated using a negative binomial generalized linear model with selection of covariates via Bayesian Information Criterion (BIC)

Covariates: ^b^alcohol use, RNA Integrity Number; ^c^gender, pH, RNA Integrity Number; ^d^gender, age, pH, RNA Integrity Number; ^e^RNA Integrity Number; ^f^alcohol use, post-mortem interval, RNA Integrity Number; ^g^alcohol use

## Discussion

Our analysis of RNA expression from the *HTR2A* gene locus provides us with valuable insight into the sense- and antisense-encoded genes of this region, and delineates the use of known and novel exons. Of note, we see evidence for expression of up to 10 distinct sense-encoded human *HTR2A* exons generated through the use of alternative transcription start sites, alternative splicing (intron retention, alternative donor site), and the infrequent use of novel splice donor/acceptor sites. On the antisense strand, we observed an abundance of novel *HTR2A-AS1* exons. Including the previously-described *HTR2A-AS1* exons, our splice junction analysis provides evidence for up to 22 unique antisense exons, 5 of which overlap with sense-encoded *HTR2A* exons (Fig. **3**a). Furthermore, we found evidence that the common SNPs rs6311 and rs6313 are associated with expression of the *HTR2A* transcripts with the extended 5’ UTR and *HTR2A-AS1* exon 14. Finally, we demonstrated that the expression of *Htr2a* and *Htr2a-AS1* in the mouse parallels expression in humans, suggesting that the mouse is suitable for studying specific sense and antisense isoforms.

### Consequences of *HTR2A* alternative splicing

The genomics era, ushered in by massively-parallel DNA sequencing, has rapidly expanded our knowledge of gene architecture. At one time, nearly all GPCRs were considered to be without introns in their open reading frames (ORFs) [37]. While GPCRs are enriched for intronless ORFs relative to other protein classes, up to 42 % of rhodopsin family GPCR genes have multiple exons that undergo alternative splicing [38]. Here, we found that *HTR2A* undergoes a variety of different splicing events, including utilization of alternative splice acceptor sites, exon skipping, rare exon usage, and intron retention, similar to previously described GPCR splicing patterns [39].

Inferred from splice junction reads, the majority of *HTR2A* transcripts encode for the full-length 7TM receptor (Table 3). However, some of the transcripts likely encode receptor proteins lacking the *N*-terminus and varying stretches of transmembrane domains; particularly the isoforms with junctions shared between exon 0 and 3 and those utilizing exon 2^tr^. The previously described 6TM form resulting from usage of exon 2^tr^ [21] bears resemblance to other GPCRs reported to have truncated or “headless” forms: *CALCR* [40], *CNR1* [41], *CCKBR* [42], *OPRL1* [43], *OPRM1* [44], and *SSTR5* [45]. While a headless form of 5-HT_2A_ would not necessarily disrupt the binding site for serotonin, it could abate membrane expression due to the deletion of *N*-glycosylation sites [46]. A comparison across species at the nucleotide level reveals strong conservation beginning specifically at the splice acceptor site for exon 2^tr^ and a relative lack of amino acid conservation in the *N*-terminus across species (Additional file 12: Figure S8). In our analysis of mouse *Htr2a*, we observed usage of the exon 2^tr^ splice site, which is predicted to result in a 7TM receptor that lacks almost the entire *N*-terminus. The *N*-glycosylation sites critical for 5-HT_2A_ membrane expression are conserved in mice, and are deleted by the use of the orthologous exon 2^tr^ splice site. Consequently, we speculate that membrane expression for the truncated receptor would be reduced in mice and humans.

It is not known whether the alternatively spliced mRNAs are translated into truncated 5-HT_2A_ protein isoforms in vivo, or subsequently capable of signaling from the cytoplasm. Assuming translation occurs, there are two observations arguing for the plausibility of intracellular 5-HT_2A_ signaling. First, serotonin that enters cells via the serotonin transporter is biologically active and involved in “serotonylation” of proteins [47]. Second, GPCRs in the cytoplasm, internalized via the endosome, can continue to signal through G protein-dependent and independent mechanisms [48]. Further studies characterizing the cell type-specific and developmental expression patterns of this truncated isoform could lend insights into the biological relevance of this, and perhaps other, headless GPCRs.

### *HTR2A-AS1*, a lncRNA

Long non-coding RNAs are stringently defined by their length (>200 nt), lack of protein-coding potential, use of independent transcriptional units, presence of canonical splice site signals, and ability to undergo alternative splicing [49]. Some lncRNAs can have over 100 exons (*SNURF-SNRPN*) [50] and many are species-specific [51, 52]. With the additional exons and junctions we uncovered here, we reassessed classification of *HTR2A-AS1*, firmly concluding that it meets criteria as an lncRNA.

The question of *HTR2A-AS1* function remains. lncRNAs have diverse roles, but most characterized up to this point influence transcription and processing of RNAs from nearby genes, sometimes by direct RNA-RNA interactions, and other times through epigenetic modifications [53]. The fact that *HTR2A-AS1* has exons directly overlapping with *HTR2A* transcripts (Fig. 3a), a feature which is also apparent in the mouse (Fig. 3b), argues strongly for the possibility of direct interaction through complementary nucleotide base-pairing, assuming the sense- and antisense-encoded transcripts are expressed at the same time and location. Direct RNA-RNA interactions could influence RNA processing, protein translation, or both. The relatively low abundance of the antisense transcripts relative to the sense isoforms and their apparent isoform diversity argues against a role in translation of the major *HTR2A* protein isoform, as these transcripts would have to exit the nucleus, find their respective targets, and impact the function of the translating ribosome.

Instead, we speculate that *HTR2A-AS1* is more likely to have roles in *HTR2A* mRNA expression or processing. The variable use of 5’ exons in *HTR2A-AS1* and *Htr2a-AS1*, coupled with their conserved overlap with alternatively-spliced *HTR2A* exons 1 and 2 (Fig. 3), could allow *HTR2A-AS1* to participate in the alternative splicing of *HTR2A* through direct RNA-RNA interactions. Additionally, the associations between the ancestral “G” allele of rs6311, greater expression of *HTR2A* exon 1ext, and decreased expression of the seemingly constitutive *HTR2A-AS1* exon 14 suggests a possible role in transcription start site usage or epigenetic modifications, perhaps through RNA-DNA interactions. In this case, we would presume that expression of the lncRNA is inversely associated with usage of the transcription start site that results in longer 5’ UTR *HTR2A* transcripts. It is also possible that *HTR2A-AS1* transcripts impact *HTR2A* expression at multiple levels and in a species-specific manner. While molecular data showing an interaction of any kind between *HTR2A-AS1* and *HTR2A* is lacking, our study provides critical information for testing these hypotheses, including evidence that an orthologous lncRNA is expressed in mice.

### *HTR2A* and *HTR2A-AS1* genetics: rs6311 and rs6313

*HTR2A* demonstrates many genotype-phentoype associations. A cursory analysis of the HuGE Navigator Genopedia shows 201 disease terms where *HTR2A* was investigated in 540 publications [54]. To put this into perspective, of the 12,488 genes listed in the HuGE Navigator, *HTR2A* ranks 58^th^ with respect to the number of associated disease terms. Of the 540 *HTR2A* publications listed, 333 specifically mention rs6311 or rs6313 (or some variation of the nomenclature; e.g. -1438 A/G, T102C, etc.) in their abstract or title, highlighting the interest in understanding disease associations conferred by these SNPs. While contradictory findings for any one phenotype-genotype association exist, overwhelming evidence implicates *HTR2A*, and specifically rs6311 or rs6313, in a variety of phenotypes.

Because rs6311 and rs6313 are in near perfect LD, genetic association studies have the liberty of using either SNP to obtain similar results. However, each SNP must be considered separately when attempting to elucidate their biological functions and contributions to disease. To date, the most compelling evidence for modulating biological function supports a role for rs6311 in the expression of *HTR2A* mRNAs with the extended 5’ UTR [21, 29], while no apparent role has been identified for rs6313. The allelic expression analysis of rs6311 in our current study supports the conclusion that rs6311 modulates expression of the extended 5’ UTR. Aside from the infrequent allelic expression imbalances noted for rs6313, we find little evidence that expression of the highly-expressed primary *HTR2A* transcript not containing the extended 5’ UTR is modulated by these common SNPs.

Regarding the transcription factors binding to the region of DNA harboring rs6311 to exert an allelic effect, the question remains open. An interesting candidate that unites genetic findings in schizophrenia is Early Growth Response 3 (*EGR3*). Polymorphisms in the promoter of the gene encoding *EGR3* are implicated in schizophrenia [55]. Multiple studies have now demonstrated a link between *EGR3*, 5-HT_2A_ receptor expression, and schizophrenia-like behaviors in transgenic animals [56, 57]. The consensus binding site for *EGR3* does align, although imperfectly, at the rs6311 region and the minor allele for rs6311 impacts this alignment (Additional file 13: Figure S9). Other transcription factors predicted to bind at the rs6311 locus *in silico* include the nuclear factor 1 family (NFIA/B/C), the Thing1/e47 heterodimer, and SMAD3 (Additional file 13: Figure S9). However, molecular evidence and additional bioinformatic studies are required to test such relationships, given the imprecision of transcription factor binding site tools in predicting relationships in vivo.

Changes in expression conferred by rs6311 or rs6313 could also result from other RNA processing events, such as alternative splicing. Considering the extent of alternative splicing on both the sense and antisense strands adjacent to rs6313, and the proximity of this SNP to alternative exons, we find it possible that this SNP could impact splicing. *In silico* analysis of splicing factors using a number of tools suggest this SNP can impact their binding (Additional file 14: Table S6). However, the aggregated results do not demonstrate a clear consensus for any single splicing factor being impacted by rs6313.

Future studies of *HTR2A-AS1* expression should consider regulation by rs6311 or rs6313, as we found some evidence suggesting differential expression across genotype. However, the low read coverage across the entire *HTR2A-AS1* transcript leads us to interpret these findings with caution. Both SNPs sit in intronic regions of *HTR2A-AS1*; rs6313 is 61 nucleotides downstream of exon 8 and rs6311 is 100 nucleotides downstream of exon 9. Both exons 8 and 9 share a relatively high number of splice junctions with the antisense exon 14 that exhibits genotype-expression associations. Our analyses are unable to isolate the individual contributions of either SNP due to high LD. Until stronger evidence delineates specific roles for each SNP, it remains equally plausible that either could modulate *HTR2A-AS1* expression.

## Conclusions

We examined *HTR2A* and *HTR2A-AS1* gene structure by mapping RNA-Seq junction reads from human prefrontal cortex, utilizing conventional definitions for exon structure independent of established gene annotations. This approach revealed two novel exons in *HTR2A* and showed the extent to which all exons are included in the mature mRNA transcripts. While the majority of transcripts consist of the previously-annotated exons that encode for the full-length 7TM protein, we also found evidence supporting the use of unannotated but previously-described exons that likely form truncated protein isoforms of 5-HT_2A_. In contrast, *HTR2A-AS1* has numerous novel exons, some of which overlap with annotated *HTR2A* exons. The biological significance of *HTR2A-AS1* transcripts is yet to be revealed, but the current findings enable studies to directly test if they regulate *HTR2A* expression. To that end, we conclude that the mouse could serve as a suitable model organism for studying whether sense-antisense interactions occur in *Htr2a*, as it also expresses many orthologous sense and antisense transcripts, and the overlapping features between the transcripts are spatially similar. Finally, we found evidence that the common genetic variant rs6311 regulates expression of *HTR2A* transcripts containing the extended 5’ UTR, consistent with previous studies. We also found associations between rs6311, rs6313, and expression of *HTR2A-AS1*. Guided by these findings, we can begin to examine variable transcript expression in the *HTR2A* gene locus, especially focusing on diseases where it is implicated.

## Methods

### Human tissues

DLPFC specimens (Brodmann area 46/9) were dissected by a trained neuropathologist (T.M.H.) from brains collected post-mortem from the Office of the Chief Medical Examiner of the State of Maryland, with audiotaped informed consent from the legal next-of-kin, as approved by the Maryland Department of Health and Mental Hygiene’s Institutional Review Board, as previously described [58, 59]. Details of tissues acquisition, handling, processing, dissection, clinical characterization, diagnoses, and neuropathological examinations were also previously described [58]. Research on post-mortem samples was conducted in accordance with the U.S. Department of Health & Human Services Code of Federal Regulations (Title 45, Part 46), in which post-mortem research is distinct from human subject research and not under the purview of a local IRB (http://www.hhs.gov/ohrp/humansubjects/guidance/45cfr46.html#46.102). Samples were de-identified and include 105 schizophrenia patients and 106 normal controls; 85 African Americans and 126 Caucasians (Demographics in Table 7). In accordance with known schizophrenia traits, smoking and suicide are noticeably higher in the affected cohort.

**Table 7:** Human Tissue Demographics

		Schizophrenia (*n* = 105)	Control (*n* = 106)
Gender	Male	74.3 % (*n* = 78)	74.5 % (*n* = 79)
	Female	25.7 % (*n* = 27)	25.5 % (*n* = 27)
Race	Caucasian	60.0 % (*n* = 63)	59.4 % (*n* = 63)
	African-American	40.0 % (*n* = 42)	40.6 % (*n* = 43)
Age at Death		45.6 ± 13.8 years	45.9 ± 13.8 years
Manner of Death	Natural	59.0 % (*n* = 62)	82.1 % (*n* = 87)
	Accidental	13.3 % (*n* = 14)	8.5 % (*n* = 9)
	Suicide	26.7 % (*n* = 28)	0.0 % (*n* = 0)
	Undetermined	1.0 % (*n* = 1)	0.9 % (*n* = 1)
	No Autopsy	0.0 % (*n* = 0)	0.9 % (*n* = 1)
	Homicide	0.0 % (*n* = 0)	7.5 % (*n* = 8)
Smoking Status at Time of Death	Smoker	70.5 % (*n* = 74)	26.4 % (*n* = 28)
	Non-smoker	21.0 % (*n* = 22)	70.8 % (*n* = 75)
	Not Determined	8.6 % (*n* = 9)	2.8 % (*n* = 3)
Brain pH		6.46 ± 0.24	6.56 ± 0.26
Post-mortem interval		40.3 ± 26.6 hours	29.4 ± 14.1 hours
RNA Integrity Number		8.0 ± 0.91	8.3 ± 0.75

### Genotyping, illumina chips

Genomic DNA was extracted from cerebellar tissues (Qiagen, Valencia CA, USA) and genotyped with HumanHap650Y_v3 or Human 1 M-Duo_v3 Illumina BeadChips (Illumina, San Diego, CA, USA) according to the manufacturer’s instructions. Genotypes were called using the Illumina GenomeStudio v2010.1 software using the default settings and the chip-specific cluster files supplied by Illumina. For data analysis, SNPs had an overall missing rate <0.02 %, CAUC and AA Hardy-Weinberg Equilibrium (HWE) p-values each > = 0.001, and minor allele frequencies (MAF) > = 0.01.

### RNA extraction and quality assessment

Tissue from DLPFC was pulverized and stored at -80 °C. Total RNA was extracted from 100 mg of tissue with TRIzol Reagent (Life Technologies, Grand Island, New York). The yield of total RNA was determined by spectrophotometry by measuring absorbance at 260 nm. RNA quality was assessed with high-resolution capillary electrophoresis on an Agilent Bioanalyzer 2100 (Agilent Technologies, Palo Alto, California), yielding an RNA Integrity Number (RIN; scale 1–10, with 1 being the lowest and 10 being the highest RNA quality).

### RNA-Seq library construction

RNA-seq libraries were constructed using Illumina TruSeq RNA sample Prep Kit, following the manufacturer’s protocol. The poly-A containing mRNA molecules were purified from ~ 800 ng DNAse treated total RNA. Following poly-A purification, the mRNA was fragmented into small pieces using divalent cations under elevated temperature (94°) for 2 min. Under this condition, fragment lengths range from 130 to 290 bp with a median length of 185 bp. Fragmented RNA was reverse transcribed into a first strand cDNA-RNA hybrid using random hexamers. Following, DNA Polymerase I and RNaseH were used to generate the complementary second strand cDNA. These double-stranded cDNA fragments then underwent end repair using T4 DNA polymerase, T4 PNK and Klenow DNA polymerase. Illumina PE barcoded adapters were ligated using T4 DNA Ligase following the addition of a single ‘A’ base using Klenow exo (3’ to 5’ exo minus). These products were then purified and PCR-enriched to create the final cDNA library for high through put DNA sequencing using a Highseq2000. The concentrations of RNA-seq libraries were measured by Qubit (Invitrogen, CA). The quality of each RNA-seq library was measured by LabChipGX (Caliper, MA) using HT DNA 1 K/12 K/HiSens Labchip.

### Mouse tissues and sequencing

All mouse data was obtained from public sources. Mouse tissues collection, RNA isolation, library preparation, and RNA-Seq were performed as described in their original publications [34–36]. Collected animal data conformed to local institutional review boards, as described in their original publications [34–36]. Raw BAM files from each of the three studies (Series Numbers GSE27243, GSE52564, and GSE36025) were downloaded from GEO (http://www.ncbi.nlm.nih.gov/geo/) and aligned with GSNAP as described below to *Mus musculus* reference genome mm10.

### RNA sequence mapping

Demultiplexing was performed with CASAVA v1.8.2 (http://support.illumina.com/sequencing/sequencing_software/casava.ilmn). Alignments were first performed with Tophat v2.0.4 [60] using the reference genome: Illumina UCSC hg19; and gene annotations: Ensembl GRCh37.67. An example command is: tophat –p 4 –r 160 –G Homo_sapiens.GRCh37.67.gtf –o Sample_out. Reads over the *HTR2A* region were extracted post processing with samtools v0.1.18 [61] with the following example command: samtools view –h Sample_RNA/accepted_hits.bam chr13:47357513-47521169 > HTR2A_regions/Sample_RNA/accepted_hits.sam. The extracted reads for each of the 211 samples were subsequently re-aligned to the human genome reference with GSNAP [62] which allows for gapped alignments, including intron-spanning alignments. Descriptive and statistical analyses were performed using this subsequent alignment.

### Alignment to the *HTR2A* locus

On average, we generated 114 million reads per sample, 82 % of which mapped to the reference genome (mapping statistics in Additional file 15: Table S1). *HTR2A* is on the reverse strand of chromosome 13. Consequently, we defined canonical splice junctions with respect to transcription on the reverse strand and subsequently mapped junction reads across putative exons in order to define exon boundaries. In order for reads at novel junctions to be attributed to *HTR2A*, they must follow canonical splicing rules such that the nucleotides comprising the presumptive splice donor and acceptor sites are GU and AG, respectively. Adherence to this rule establishes the genomic DNA strand from which transcription occurred, eliminating ambiguity when assigning junction reads to *HTR2A* versus the overlapping *HTR2A-AS1*. This resulted in an average of 7248 aligned reads per sample over the *HTR2A* locus. Observational analysis, for the purpose of correcting for obvious bias in *HTR2A* mapping, revealed no overt differences across diagnosis or sex with respect to *HTR2A* expression.

### RNA quantification

Gene expression was quantified using the total number of reads for each sample that uniquely aligned to the reference. The read depth of each gene was computed based on the coordinates of mapped reads and newly annotated exons in the reference genome. Visualization of the mapping was carried out using publicly available software, primarily the Integrated Genomic Viewer (IGV) (http://www.broadinstitute.org/software/igv/).

### Allelic expression imbalance

Allele-specific expression was assessed for those samples that were heterozygous over rs6311 and rs6313. For measuring quality base counts, we used the following command, varying the region and sample: samtools mpileup -uD -r chr13:47357513-47521169 -f ref_genome.fa R3924_*.bam | bcftools view -cg - | grep 47469940. Read counts from the DP4 field was used in a binomial statistical test with a 5 % multiple test corrected significance cut-off.

### *In silico* splicing and transcription factor binding analysis

Sequences 100 bp upstream and downstream of exons was downloaded from UCSC Genome Browser and submitted to the Human Splice Finder v2.4.1 *in silico* webserver (http://www.umd.be/HSF/), which scores splicing characteristics (splice donor site, acceptor site, branch point) [30]. For the branch point analysis, the highest scoring motif between positions -50 and -17 relative to the exon start site was considered for analysis. Positions -40 to -5 were analyzed for polypyrimidine frequency, expressed as a percentage.

Splicing characteristics were also scored using the Alternative Splice Site Predictor [31]. For this analysis, sequence for the entire *HTR2A* region was downloaded via the UCSC Genome Browser and submitted to the webserver (http://wangcomputing.com/assp/index.html) for scoring. We compared the average of scores obtained for annotated RefSeq exons vs. newly-discovered exons vs. all other predicted splice donor/acceptor sites using independent sample t-tests. In-depth analysis of splicing events for rs6313 alleles used multiple *in silico* tools for predicting putative splicing-related proteins [63–67].

Transcription factor binding for rs6311 alleles was performed using MatInspector (http://www.genomatix.de/matinspector) [68].

### Statistical methods

A natural choice to model read counts is the Poisson distribution. However, it has been shown that Poisson distribution does not capture biological variation in read counts [69–71]. To account for the extra variation found in biological replicates, the Poisson distribution can be extended through the negative binomial distribution, which has been used extensively to model RNA-Seq data [72, 73]. Thus, a generalized linear model (GLM) with logit link function using the negative binomial distribution was used to analyze read counts. The genotypic model was log(UniquelyMappedReads*ExpressionRate) = Covariates + SNP. The best set of covariates was chosen using the model that minimized the Bayesian Information Criterion. Covariates considered were age, race, gender, diagnosis, alcohol use, smoking status, PMI, brain pH, RIN, and suicide. The brain pH and RIN were the main covariates explaining significant amounts of read count variability and were included in most models. Multiplicity correction was performed using a Bonferroni adjustment for the number of endpoints tested times the effective number of independent SNPs tested [74], to control type I error at an experiment-wise alpha level of 0.2.

## Availability of supporting data

Human DLPFC RNA-Seq and genotyping data will be available for download in early 2016 according to the data sharing policy described Schubert et al. (2015) [75]. Mouse data used in this study can be downloaded from GEO at http://www.ncbi.nlm.nih.gov/geo/query/acc.cgi?acc=GSE27243 [34], http://www.ncbi.nlm.nih.gov/geo/query/acc.cgi?acc=GSE52564 [35], and http://www.ncbi.nlm.nih.gov/geo/query/acc.cgi?acc=GSE36025 [36]. Mapping software used in the current study includes CASAVA 1.8.2 (http://support.illumina.com/sequencing/sequencing_software/casava.ilmn), TopHat 2.0.4 (https://ccb.jhu.edu/software/tophat/index.shtml) [60], samtools v0.1.18 (http://samtools.sourceforge.net/) [61], and GSNAP (http://research-pub.gene.com/gmap/) [62]. Aligned .BAM files were viewed using IGV (https://www.broadinstitute.org/igv/). Splicing analyses were performed using Human Splice Finder v2.4.1 (http://www.umd.be/HSF/) [30], Alternative Splice Site Predictor (http://wangcomputing.com/assp/index.html) [31], SpliceAid2 (http://www.introni.it/splicing.html) [63], Rescue-ESE (http://genes.mit.edu/burgelab/rescue-ese/) [64], FAS-ESS (http://genes.mit.edu/fas-ess/) [65], SFmap (http://sfmap.technion.ac.il) [66], ACEScan (http://genes.mit.edu/acescan2/) [67]. Transcription factor analysis was performed using MatInspector (http://www.genomatix.de/matinspector) [68].

## Additional files

Additional file 1: Figure S1. Revised gene model for human *HTR2A. (PDF 112 kb)*
Additional file 2: Figure S2. Predicted splice donor, splice acceptor, branch site, and polypyrimidine tract scores for annotated vs. novel *HTR2A* exons. (PDF 144 kb) Additional file 3: Figure S3. Reads mapped at the 5’UTR of human *HTR2A* and predicted transcription start sites, visualized using IGV. (PDF 102 kb) Additional file 4: Figure S4. Reads mapped at the 3’UTR of human *HTR2A* and predicted polyadenylation signals, visualized using IGV. (PDF 108 kb) Additional file 5: Figure S5. Reads mapped at exon 3b of human *HTR2A* and predicted polyadenylation signals, visualized using IGV. (PDF 105 kb) Additional file 6: Table S2. Splice junctions and counts for human *HTR2A-AS1. (XLSX 12 kb)*
Additional file 7: Table S3. Predicted *HTR2A-AS1* transcripts and open reading frames. (XLSX 13 kb) Additional file 8: Table S4. Mouse *Htr2a* exons and coordinates. (XLSX 12 kb) Additional file 9: Figure S6. Revised gene model for mouse *Htr2a. (PDF 93 kb)*
Additional file 10: Figure S7. Reads mapped at the 3’UTR of mouse *Htr2a* and predicted polyadenylation signals, visualized using IGV. (PDF 101 kb) Additional file 11: Table S5. Mouse *Htr2a-AS1* exons and coordinates. (XLSX 12 kb) Additional file 12: Figure S8. Predicted conservation of 5-HT_2A_ protein across 9 species. (PDF 165 kb) Additional file 13: Figure S9. Putative transcription factor binding sites at the rs6311 locus in human *HTR2A. (PDF 106 kb)*
Additional file 14: Table S6. Predicted splicing proteins bound to human *HTR2A* rs6313 alleles. (XLSX 11 kb) Additional file 15: Table S1. Mapping statistics for human DLPFC RNA-Seq samples (XLSX 22 kb)
