# Supplementary material for: Genomic structure and expression of the human serotonin 2A receptor gene (HTR2A) locus: identification of novel HTR2A and antisense (HTR2A-AS1) exons
Source: BMC Genet. 2016 Jan 6;17:16. doi: 10.1186/s12863-015-0325-6 (PMC4702415; doi:10.1186/s12863-015-0325-6)
Supplement: Additional file 1: Figure S1. — Revised gene model for human HTR2A. (PDF 112 kb) [file 12863_2015_325_MOESM1_ESM.pdf]

Supplemental Figures

Figure S1 – Revised gene model for human *HTR2A*.

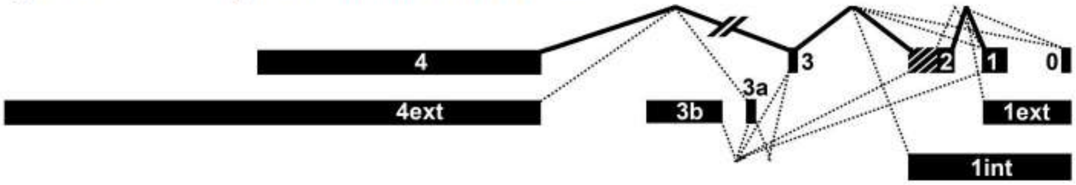

Figure 1. Human *HTR2A* gene structure. Exons are represented by black bars. The hatched portion of exon 2 represents exon 2tr. Common splice junctions are represented by solid, while dotted lines represent less common splice junctions. Refer to Table 2 for precise genomic coordinates and Table 3 for splice junction frequencies. *Note:* gene is encoded 3′-to-5′ from left-to-right.
