# Supplementary material for: Genomic structure and expression of the human serotonin 2A receptor gene (HTR2A) locus: identification of novel HTR2A and antisense (HTR2A-AS1) exons
Source: BMC Genet. 2016 Jan 6;17:16. doi: 10.1186/s12863-015-0325-6 (PMC4702415; doi:10.1186/s12863-015-0325-6)
Supplement: Additional file 2: Figure S2. — Predicted splice donor, splice acceptor, branch site, and polypyrimidine tract scores for annotated vs. novel HTR2A exons. (PDF 144 kb) [file 12863_2015_325_MOESM2_ESM.pdf]

Figure S2 – *HTR2A* Exon Characteristics.

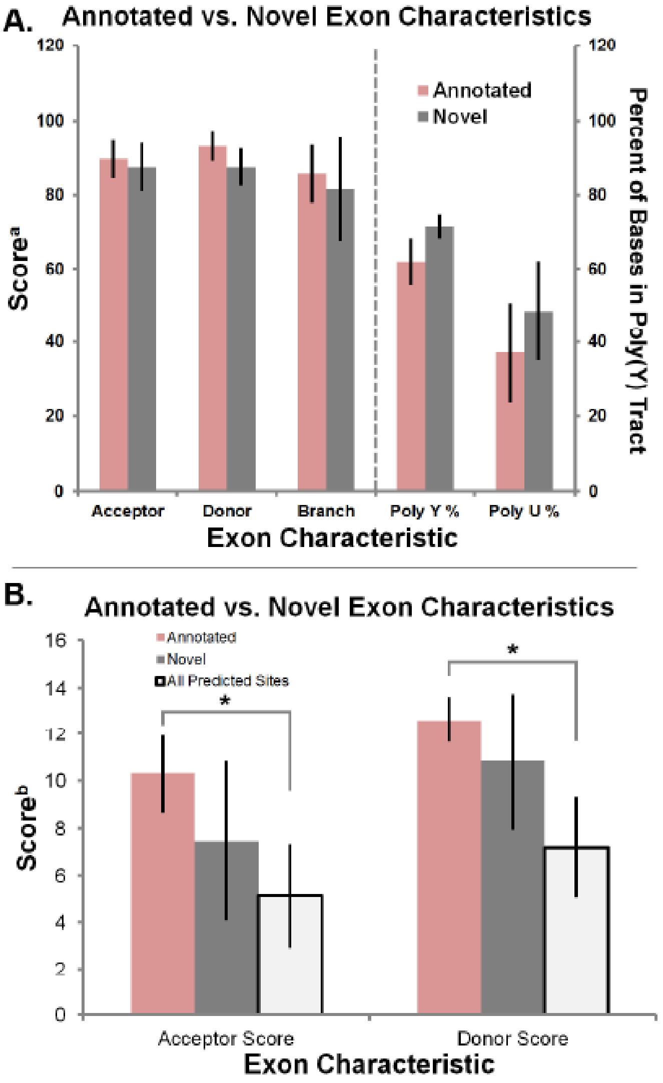

Figure S2. ***(A)*** Splice acceptor, splice donor, and branch site scores did not significantly differ across annotated and novel exons in *HTR2A*. The enrichment of polypyrimidines [Poly(Y)] in the Poly(Y) tract, defined as bases -5 to -40 upstream of the exon, also did not significantly differ. ***(B)*** Using a different *in silico* prediction program, we again see no significant difference between splice acceptor or splice donor scores for annotated versus novel exons. On average, annotated exons did score significantly higher than the all predicted sites in the *HTR2A* gene locus (*p<0.05). [a]Scores predicted by the Human Splicing Finder (http://www.umd.be/HSF/; Desmet et al., 2009). [b]Scores predicted by the Alternative Splice Site Predictor (http://wangcomputing.com/assp/index.html; Wang et al., 2006). Error bars depict standard deviation.
