# Supplementary material for: Genomic structure and expression of the human serotonin 2A receptor gene (HTR2A) locus: identification of novel HTR2A and antisense (HTR2A-AS1) exons
Source: BMC Genet. 2016 Jan 6;17:16. doi: 10.1186/s12863-015-0325-6 (PMC4702415; doi:10.1186/s12863-015-0325-6)
Supplement: Additional file 3: Figure S3. — Reads mapped at the 5’UTR of human HTR2A and predicted transcription start sites, visualized using IGV. (PDF 102 kb) [file 12863_2015_325_MOESM3_ESM.pdf]

Figure S3 – Mapped Reads in Human *HTR2A* 5' UTR

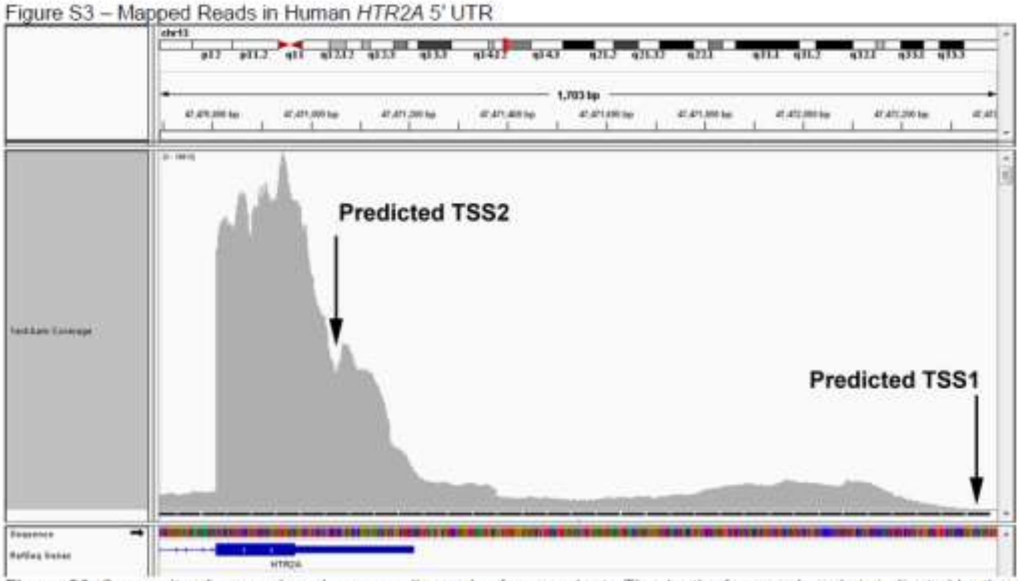

Figure S3. Composite of mapped reads across all samples for exon 1ext. The depth of mapped reads is indicated by the gray histogram in the upper panel, while *HTR2A* gene structure is depicted in the lower panel (3'-to-5' direction from left-to-right). The histogram suggests the presence of at least two different transcription start sites (arrows), which coincide with predicted sites from Eponine (Down & Hubbard, 2002). *Note*: read depth is presented in linear scale.
