# Supplementary material for: Genomic structure and expression of the human serotonin 2A receptor gene (HTR2A) locus: identification of novel HTR2A and antisense (HTR2A-AS1) exons
Source: BMC Genet. 2016 Jan 6;17:16. doi: 10.1186/s12863-015-0325-6 (PMC4702415; doi:10.1186/s12863-015-0325-6)
Supplement: Additional file 4: Figure S4. — Reads mapped at the 3’UTR of human HTR2A and predicted polyadenylation signals, visualized using IGV. (PDF 108 kb) [file 12863_2015_325_MOESM4_ESM.pdf]

Figure S4 – Extension of Human *HTR2A* 3′ Untranslated Region

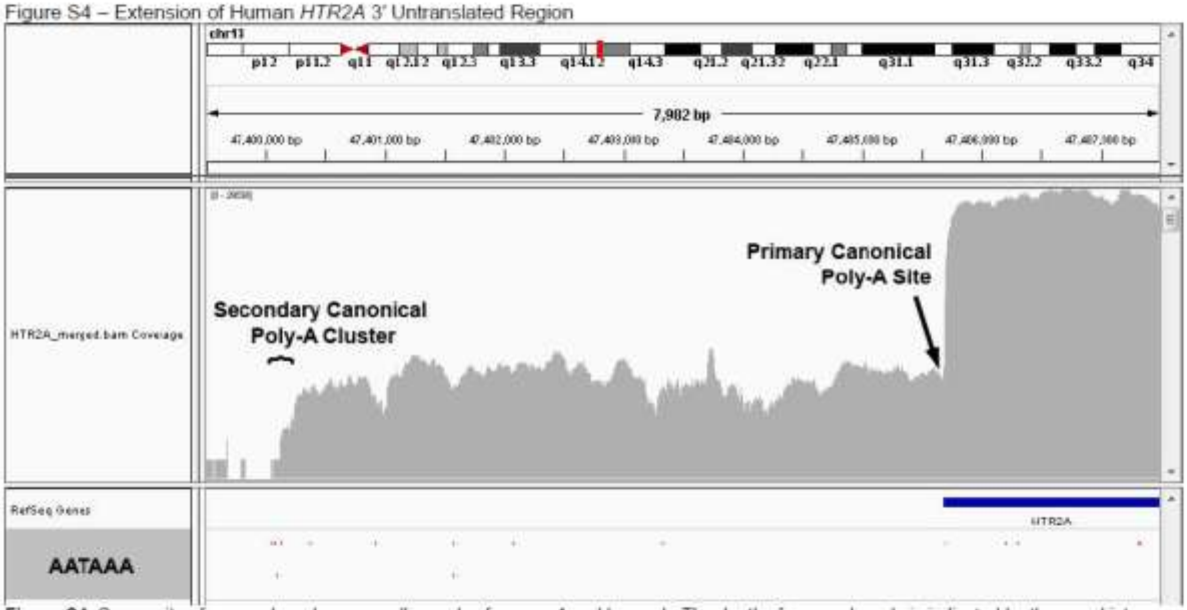

Figure S4. Composite of mapped reads across all samples for exon 4 and beyond. The depth of mapped reads is indicated by the gray histogram in the upper panel, while *HTR2A* gene structure is depicted in the lower panel (3′-to-5′ direction from left-to-right). The histogram suggests an extension of the 3′ UTR beyond the canonical poly-A site at the terminus of the annotated exon 4, continuing to a cluster of poly-A signals approximately 5.6kB distal to *HTR2A*. Transcripts with this greatly extended 3′ UTR constitute a very small percentage (<1%) of reads relative to the annotated 3′ UTR. *Note*: read depth is presented in $\log_{10}$ scale.
