# Supplementary material for: Genomic structure and expression of the human serotonin 2A receptor gene (HTR2A) locus: identification of novel HTR2A and antisense (HTR2A-AS1) exons
Source: BMC Genet. 2016 Jan 6;17:16. doi: 10.1186/s12863-015-0325-6 (PMC4702415; doi:10.1186/s12863-015-0325-6)
Supplement: Additional file 5: Figure S5. — Reads mapped at exon 3b of human HTR2A and predicted polyadenylation signals, visualized using IGV. (PDF 105 kb) [file 12863_2015_325_MOESM5_ESM.pdf]

Figure S5 – Human Exon 3b Coverage and Predicted Poly-A Signals

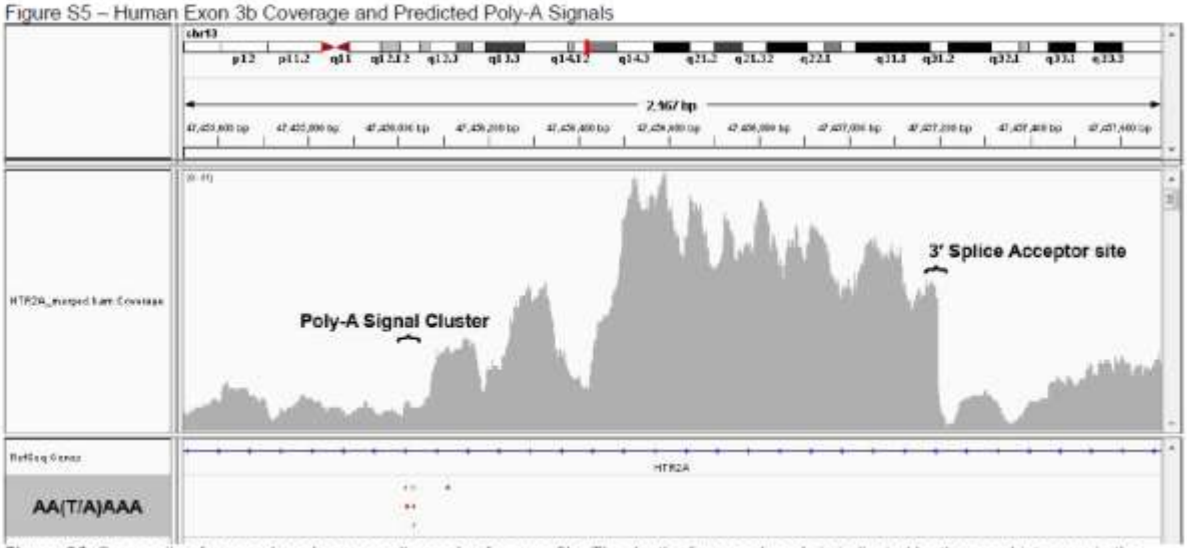

Figure S5. Composite of mapped reads across all samples for exon 3b. The depth of mapped reads is indicated by the gray histogram in the upper panel, while *HTR2A* gene structure is depicted in the lower panel (3'-to-5' direction from left-to-right). The histogram depicts exon 3b starting adjacent to the 3' splice acceptor site and continuing approximately 1.2kB to the poly-A signal cluster, proximal to where read depth noticeably decreases. Canonical and non-canonical poly-A sites are marked in the lower panel as red dots. *Note*: read depth is presented in linear scale.
