# Supplementary material for: Genomic structure and expression of the human serotonin 2A receptor gene (HTR2A) locus: identification of novel HTR2A and antisense (HTR2A-AS1) exons
Source: BMC Genet. 2016 Jan 6;17:16. doi: 10.1186/s12863-015-0325-6 (PMC4702415; doi:10.1186/s12863-015-0325-6)
Supplement: Additional file 9: Figure S6. — Revised gene model for mouse Htr2a. (PDF 93 kb) [file 12863_2015_325_MOESM9_ESM.pdf]

Figure S6 – Revised Gene Model and Splicing for Mouse *Htr2a*

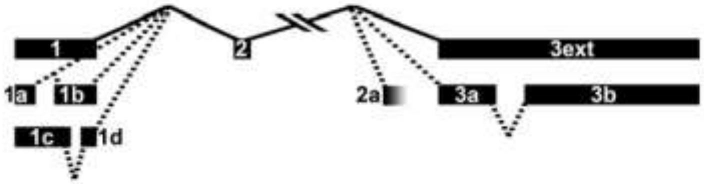

Figure S5. Mouse *Htr2a* gene structure. Exons are represented by black bars. The 3' end of exon 2a is undefined. Splicing events are represented by lines (solid = common splicing event, dotted = less common splicing event). Exons 1a, 1b, 2 and 3ext are orthologous to human exons 1, 2, 3, and 4, respectively. Exon 1d is orthologous to human exon 2tr. We did not find exons orthologous to 1c, 2a, 3a, or 3b in humans. Refer to Table S4 for precise genomic coordinates. *Note*: gene is encoded 5'-to-3' from left-to-right.
