# Supplementary material for: Genomic structure and expression of the human serotonin 2A receptor gene (HTR2A) locus: identification of novel HTR2A and antisense (HTR2A-AS1) exons
Source: BMC Genet. 2016 Jan 6;17:16. doi: 10.1186/s12863-015-0325-6 (PMC4702415; doi:10.1186/s12863-015-0325-6)
Supplement: Additional file 10: Figure S7. — Reads mapped at the 3’UTR of mouse Htr2a and predicted polyadenylation signals, visualized using IGV. (PDF 101 kb) [file 12863_2015_325_MOESM10_ESM.pdf]

Figure S7 – Mouse 3' UTR Coverage and Poly-A Signals

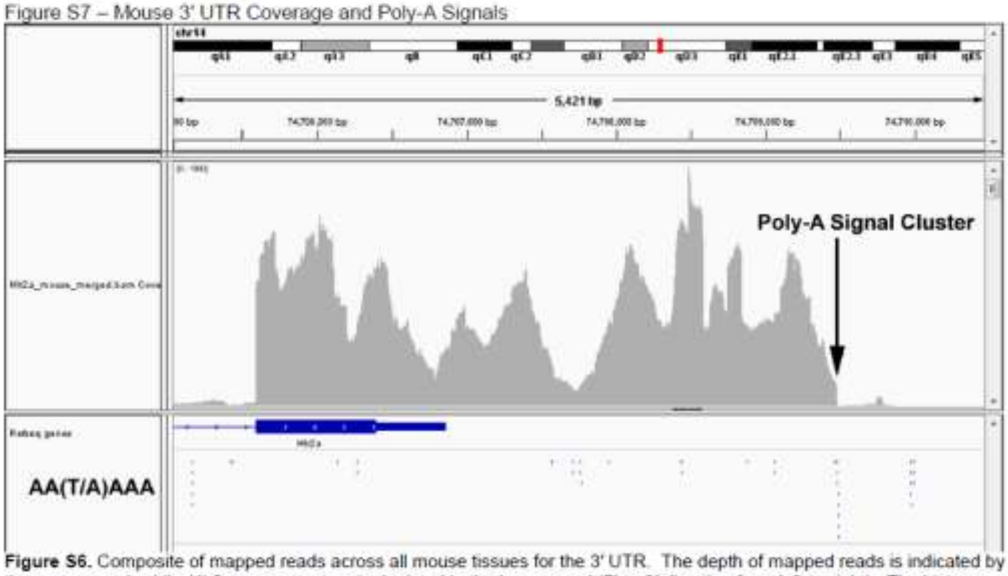

Figure S6. Composite of mapped reads across all mouse tissues for the 3' UTR. The depth of mapped reads is indicated by the gray histogram in the upper panel, while *Htr2a* gene structure is depicted in the lower panel (5'-to-3' direction from left-to-right). The histogram depicts reads extending approximately 2.6 kB beyond the annotated terminus of *Htr2a*, continuing to a cluster of canonical and non-canonical poly-A signals (blue dots in lower panel). *Note*: read depth is presented in linear scale.
