# Supplementary material for: Genomic structure and expression of the human serotonin 2A receptor gene (HTR2A) locus: identification of novel HTR2A and antisense (HTR2A-AS1) exons
Source: BMC Genet. 2016 Jan 6;17:16. doi: 10.1186/s12863-015-0325-6 (PMC4702415; doi:10.1186/s12863-015-0325-6)
Supplement: Additional file 12: Figure S8. — Predicted conservation of 5-HT2A protein across 9 species. (PDF 165 kb) [file 12863_2015_325_MOESM12_ESM.pdf]

Figure S8 – Species Conservation of 5-HT_{2A} Protein

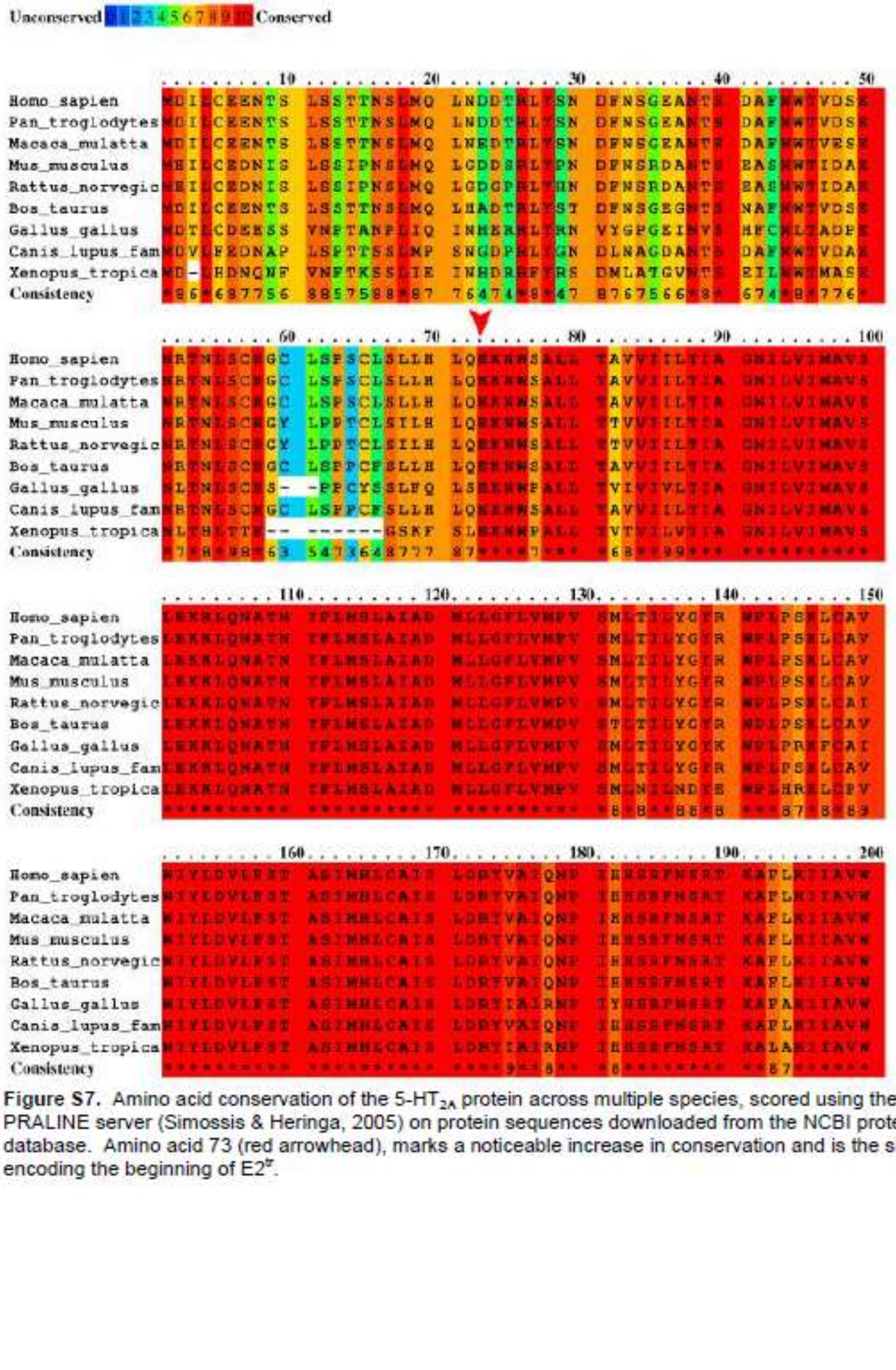

Figure S7. Amino acid conservation of the 5-HT_{2A} protein across multiple species, scored using the PRALINE server (Simossis & Heringa, 2005) on protein sequences downloaded from the NCBI protein database. Amino acid 73 (red arrowhead), marks a noticeable increase in conservation and is the site encoding the beginning of $E2^{tr}$.
