# Supplementary material for: Genomic structure and expression of the human serotonin 2A receptor gene (HTR2A) locus: identification of novel HTR2A and antisense (HTR2A-AS1) exons
Source: BMC Genet. 2016 Jan 6;17:16. doi: 10.1186/s12863-015-0325-6 (PMC4702415; doi:10.1186/s12863-015-0325-6)
Supplement: Additional file 13: Figure S9. — Putative transcription factor binding sites at the rs6311 locus in human HTR2A. (PDF 106 kb) [file 12863_2015_325_MOESM13_ESM.pdf]

Figure S9 – Transcription Factor Binding for rs6311

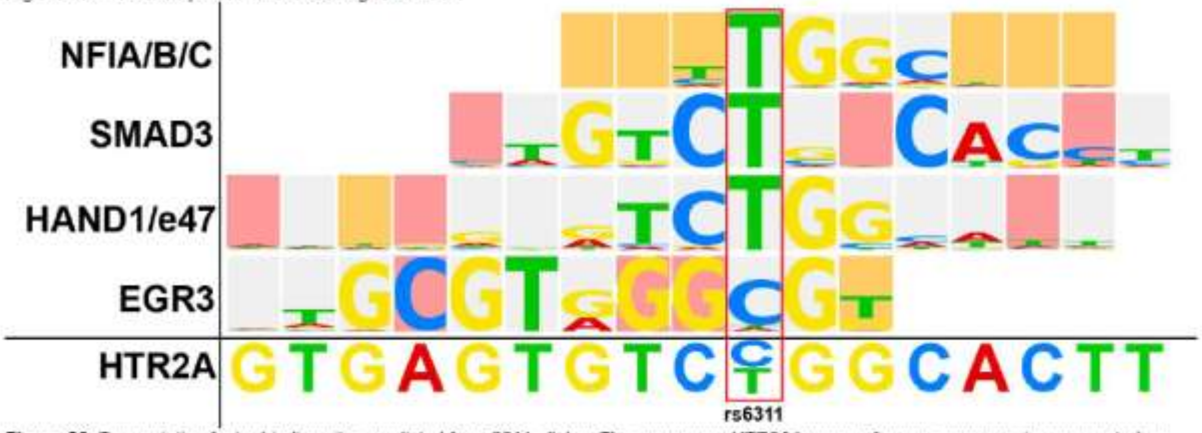

Figure S8. Transcription factor binding sites predicted for rs6311 alleles. The consensus *HTR2A* human reference sequence is represented on the bottom row, with the predicted transcription factor alignments above. The bi-allelic rs6311 position is denoted by the red box. Weak matches, where the second nucleotide in the transcription factor prediction weight matrix constitutes >20% of the total weight, are shaded orange, while mismatches (<20%) are shaded pink.
